# Male Facial Appearance and Offspring Mortality in Two Traditional Societies

**DOI:** 10.1371/journal.pone.0169181

**Published:** 2017-01-12

**Authors:** Lynda G. Boothroyd, Alan W. Gray, Thomas N. Headland, Ray T. Uehara, David Waynforth, D. Michael Burt, Nicholas Pound

**Affiliations:** 1 Department of Psychology, Durham University, Durham, United Kingdom; 2 Department of Anthropology, SIL International, Dallas, Texas, United States of America; 3 Faculty of Health Sciences & Medicine, Bond University, Queensland, Australia; 4 Division of Psychology Department of Life Sciences, Brunel University London, Uxbridge, United Kingdom; Macquarie University, AUSTRALIA

## Abstract

It has been hypothesised that facial traits such as masculinity and a healthy appearance may indicate heritable qualities in males (e.g. immunocompetence) and that, consequently, female preferences for such traits may function to increase offspring viability and health. However, the putative link between paternal facial features and offspring health has not previously been tested empirically in humans. Here we present data from two traditional societies with little or no access to modern medicine and family planning technologies. Data on offspring number and offspring survival were analysed for the Agta of the Philippines and the Maya of Belize, and archive facial photographs were assessed by observers for attractiveness and masculinity. While there was no association between attractiveness and offspring survival in either population, a quadratic relationship was observed between masculinity and offspring survival in both populations, such that intermediate levels of masculinity were associated with the lowest offspring mortality, with both high and low levels of masculinity being associated with increased mortality. Neither attractiveness nor masculinity were related to fertility (offspring number) in either population. We consider how these data may or may not reconcile with current theories of female preferences for masculinity in male faces and argue that further research and replication in other traditional societies should be a key priority for the field.

## Introduction

Human females appear to show systematic variation in their preferences for putative craniofacial indicators of testosterone exposure in males (i.e. facial masculinization). Research in this area typically involves assessing variation in female preferences either for faces that are perceived as masculine, or variation in preferences for faces in which the degree of sexual dimorphism has been measured, or varied, objectively. In general, preferences for masculinity appear to be stronger when females are asked to assess partners for short-term sexual relationships rather than for long-term pair-bonds [1–3]. Moreover, masculinity preferences appear stronger during the more fertile phases of the menstrual cycle [4].

This pattern of variation, in particular differences between preferences in short-term vs. long-term contexts, has been interpreted as reflecting females making trade-offs between cues to male willingness to supply paternal investment and cues to “genetic quality” [e.g. 5]. According to this perspective, male facial masculinity is considered to signal immunocompetence given the assumed immunosuppressive effects of testosterone (for a review see [6]). That is, facial masculinity is proposed to be an honest signal of heritable immunity to local pathogens (*sensu* Folstad & Karter [7]) and consequently an indicator of “good genes” which could provide indirect fitness benefits to female mates through enhancing the viability and health of offspring. However, the need for trade-offs arises as a consequence of masculinity’s assumed association with reduced parental investment; more masculine men are perceived as worse parents and less committed partners [8], and testosterone may likewise be associated with relationship and paternal investment (for discussion see [9]).

Evidence for this immunocompetence-based account of variation in female preferences, however, remains largely tangential. Although research has suggested at least one candidate route for testosterone to affect immune responses via gene modulation [10], the literature on links between testosterone and immunity in humans and other species remains complex and somewhat inconsistent (for discussion see [11, 6]), and there have been very few studies examining the links between facial masculinity and health in adult men (for discussion see [6, 12]). Furthermore, although the immunocompetence hypothesis is typically discussed in the facial attraction literature as if it necessarily suggests that masculinity ought to correspond in a fairly simple, linear manner with underlying health, the original theory in fact predicts a variety of possible phenotypic relationships between signal strength (i.e. masculinity) and pathogen load [13]. The more central assumption of the hypothesis, that men with more masculine faces sire offspring that are more viable and healthy, has never been tested empirically. One study found that men with a more masculine appearance had sons that were more masculine, but not more attractive looking [14] but it is likely that in relatively affluent, contemporary Western populations, modern medicine may obscure relationships between individual characteristics and health outcomes. Ancestral human populations likely experienced substantial levels of infant and childhood mortality [15]. Consequently, of particular importance to immunocompetence-based theories of female attraction to masculine facial features, is the extent to which those features are associated with greater offspring health and survival in pre-industrial populations (see e.g. [16] for discussion of testing evolutionary hypotheses in appropriate samples).

At the time of writing, although no previous studies have examined associations between facial masculinity and offspring outcomes there has been a report that another sexually dimorphic trait (voice pitch) is associated with reproductive success in a non-Western population. Apicella and colleagues [17] found that amongst the Hadza of Tanzania, men with more masculine voices had more offspring but those offspring did not enjoy greater survival than the children of men with less masculine voices (although the correlation was in the expected direction). The relationship between paternal facial masculinity and offspring health and survival, however, remains untested. Therefore, here we examine data from two traditional societies for whom both facial photographs and genealogical data exist, allowing us to address the issues outlined above.

## Ethics

Ethical approval for the collection of facial ratings was obtained from the Durham University Department of Psychology Ethics Committee–and after receiving a verbal or written explanation of what study participation would involve, all raters provided informed consent prior to participation, indicated by clicking a button to commence the face rating task on a computer. The secondary data analyses presented here are based on photographs and genealogical data drawn from archival data sets arising from previous research projects (Agta [18,19,20]; Maya [21]) during which all those individuals photographed by TH or DW gave their verbal assent to their image being used in research. Those photographed by earlier ethnographers (Agta only) are now deceased.

## Population 1: The Agta

The Casiguran Agta are a group of egalitarian forest dwelling hunter gatherers in the Philippines who have been studied since 1962 by Headland and colleagues [18,19] with a total documented population of 609 individuals. Demographic data gathered through interviews have been compiled into the *Agta Demographic Database* [20] which is used as the source for the present study. Median total-fertility rate amongst the Agta is 7 births per female (range 0–14) with mean age at first birth of 20.6 years. The Agta are monogamous and bilocal with a strong tendency towards exogamy (see [22] for further discussion). Pair bonds amongst the Agta are relatively stable (only 15.9% of marriages, trial marriages and temporary unions in the database ended in separation) and child mortality is moderately high (approximately 28% of individuals recorded in the database died by 5 years of age). Agta infants typically co-sleep with their parents; although mothers are the primary carers, fathers provide direct care as required, typically when mothers are engaged in subsistence work (see e.g. [23], for discussion).

### Sample

Our sample was drawn from the 250 males in the *Agta Demographic Database* who were photographed between the ages of 17 and 70 years. Analyses were restricted to the 91 of these men who had fertility data that were definitely or probably accurate (i.e. were the likely fathers of all putative offspring in the database and unlikely to have sired offspring outside the community). The photographs (some colour and some black and white) were a mixture of those taken by early ethnographers, and those taken by Headland (some individual and some cropped from group photographs) for the purpose of memorising names. These individuals were born between 1912 and 1987 and photographed between 1936 and 2010.

### Offspring outcomes

For each individual in the database, current age or age at death and father’s identity was extracted. For each father, his fertility was defined as the number of his liveborn offspring recorded in the database. Child mortality was calculated as the proportion of each father’s liveborn children who died by 5 years of age; a second mortality variable, child mortality due to natural causes, was created by excluding deaths of children known to be due to accidents or violence. Where an adult male had been photographed but was not recorded as the father of any other individual in the database, he was given a fertility of zero. All individuals for whom photographs existed were coded by TH as their fertility data being ‘definitely incomplete’, ‘definitely complete’, ‘probably complete’ and ‘unknown’ (with inward/outward migration to/from the community being the most common reason for incomplete fertility data). Only those males photographed between the ages of 17 and 70 years, whose fertility data were definitely or probably complete, were utilised in the analyses, giving a sample of 91 individuals of whom 65 had sired offspring. Among these males, since total fertility was, as should be expected, positively associated (*r* = 0.530, *n* = 65, *p* < 0.0001) with father's recorded lifespan (i.e. age at death or current age for living males), residuals from this linear association were used in subsequent analyses as a measure of fertility corrected for lifespan.

### Facial appearance ratings

The faces of the full sample of 91 photographed individuals with fertility data that were accurate or probably accurate were assessed by 13 British observers (7 female, 6 male, aged 16–28) and four Filipino observers (2 female, 2 male, aged 19–25), recruited through word of mouth, who rated each face for attractiveness and masculinity on 1–7 Likert scales. All faces were front-on or turned at most to a ¾ angle; photographs were masked to show only the face (hair, ears, neck and clothes were excluded) and cropped and resized to fit the face into a 400 x 500 pixel area. British observers were tested in person, while Filipino observers completed the task across the internet; otherwise the testing procedure was the same. All observers were simply told they would be ‘rating faces on physical traits’ and none of them knew the individuals photographed. Each face was rated individually, and all faces were rated for one trait before all being rated for another. Order of presentation for faces within each trait and order of traits was randomised. One British observer whose ratings were negatively correlated with several other observers’ ratings was removed; otherwise inter-rater reliability was good in both the British (α = .83) and Filipino (α = .77) raters. British and Filipino ratings were correlated (attractiveness: *r* = .46, *p* < 0.001; masculinity: *r* = .72, *p*<0.001; *n* = 91) and so scores were calculated for each image based on the mean of all 16 ratings. Attractiveness ratings were normally distributed (Shapiro Wilk = .99, *p* = .88), while masculinity ratings showed deviation from normality (Shapiro Wilk = .92, *p* = .001). Given that the masculinity ratings were unimodal, however, and that the residuals in the regression analyses below were normally distributed, the ratings were used untransformed. None of the correlational results described below were altered by use of Spearman’s instead of Pearson’s coefficients.

Mean age at the time of being photographed was 35.2 years (± 13.3) and the age of individuals in their photographs was negatively associated with ratings of attractiveness (*r* = -.354, *n* = 91, *p* < 0.001) and positively associated with ratings of masculinity (*r* = .403, *n* = 91, *p* < 0.0001)–i.e. individuals who were older at the time of being photographed looked less attractive and more masculine. Consequently, to correct for the effects of age in the photographs linear regression analyses were carried out separately with attractiveness and masculinity as dependents and age in photo as the independent variable. Calculated residuals were then used in subsequent analyses as measures of each rated variable corrected for age at the time of being photographed.

### Facial appearance and offspring outcomes

**Fig 1:**
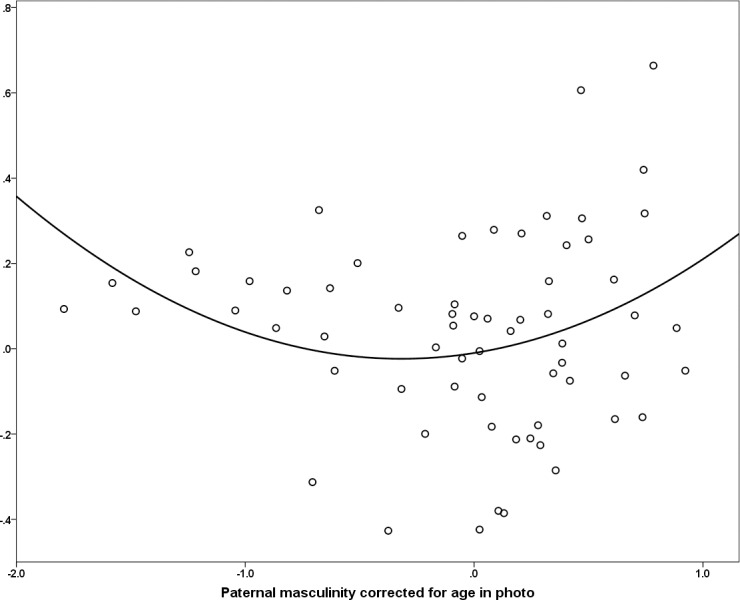
Facial masculinity and offspring mortality in Agta males. Quadratic association in Agta males [*F*(2,62) = 6.30, *p* < 0.001, *R*^2^ = .17] between facial masculinity ratings corrected for age in photo and offspring mortality due to all causes (proportion dead before 5 years of age) corrected for father’s current age or age at death if deceased. Variables are residuals from masculinity-age in photo and mortality-maximum age regression analyses.

Facial masculinity corrected for age did not correlate with attractiveness corrected for age (*r* = -0.020, *n* = 91, *p* = 0.850). Moreover, among those who had fathered liveborn offspring, there were no linear associations between offspring mortality and ratings of attractiveness (all-cause mortality: *r* = -0.016, *n* = 65, *p* = 0.90; natural causes: *r* = -0.017, *n* = 65, *p* = 0.90), or masculinity (all-cause mortality: *r* = 0.033, *n* = 65, *p* = 0.79; natural causes: *r* = 0.105, *n* = 65, *p* = 0.41) corrected for age. In addition, neither attractiveness (*r* = 0.130, *n* = 65, *p* = 0.30) nor masculinity (*r* = -0.183, *n* = 65, *p* = 0.143) were associated with fertility amongst men who had sired offspring, and fathers were no more attractive or masculine than non-fathers (see Table 1.) Inspection of scatterplots, however, suggested the existence of a non-linear relationship between masculinity and offspring mortality. Consequently, curve estimation in SPSS 20.0 was used to assess the fit of a nonlinear function and quadratic associations were found between rated masculinity and both all cause offspring mortality, [*F*(2,62) = 6.30, *p* < 0.001, *R*^2^ = .17] and childhood mortality due only to natural causes [*F*(2,62) = 6.91, *p* < 0.01, *R*^2^ = .18]. Residuals in both models were normally distributed (*p* > 0.2 for both). As shown in Fig 1, individuals with intermediate levels of masculinity exhibited lower offspring mortality than either the most or least masculine males. There were no other significant quadratic associations between facial appearance and offspring outcomes (either fertility or mortality).

**Table 1:** Differences between fathers and non-fathers in both samples.

		difference	*t*	*df*	*p*
Agta	Attractiveness^i^	0.08	0.88	89	.38
	Masculinity^i^	0.13	0.20	89	.40
	Age	-13.39	-3.39	89	.001
Maya	Attractiveness^i^	-0.33	-1.59	28	.12
	Masculinity^i^	-0.25	-0.79	28	.43
	Age	-11.97	-1.75	43	.09
	Height	-0.53	-0.69	43	.49
	Income ($)	-440	-0.254	43	.80
	# partners^i^	-0.28	0.35	43	.53

^i^ corrected for age.

## Population 2: The Maya

Waynforth recruited men aged between 18 and 72 years from two villages in western Belize in 1997 [21] by sampling from 10% of households (total population of both villages was 1500). Sixty-three percent of the men identified themselves as Mayan, 34% were Mayan through one parent only or were Mestizo, and 3% belonged to other ethnic groups. The villagers engaged in a mixture of slash-and-burn agriculture (48% of sample) or in paid labour. Like the Agta, relationships amongst the Maya tended to be relatively stable: 84% of the 56 men in the sample lived with both parents for their entire childhood [21]. Most marriages were arranged by the family, with patrilocal and neolocal residence; all men were monogamous in the current data. Mean number of offspring was 4.2 (range of 0–17) and mean age at marriage for the men was 24.2. Child mortality was high by modern industrialized population standards, but fairly low for a traditional society with little access to modern medical care: 7.7% of live-born children in the sample died by age five. The causes of child mortality were primarily infectious disease including malaria, dengue, dysentery and yellow fever.

### Sample

Of the 56 males between the ages of 18 and 75 who were interviewed by DW, fertility data were available for 45 individuals, 38 of these had fathered offspring and offspring mortality data were available for 36 of these. Photographs were taken of 35 of the men at the time of data collection (photographs of the other males were taken but the film was damaged during development) and of those 35, 23 (65.7%) had fathered offspring. Additional available data included number of wives and lifetime number of sexual partners, and total annual income derived from paid work and sale of farm produce (Belizean dollars). For statistical tests, where data were missing pairwise deletion was used to maintain power.

### Offspring outcomes

For each participant, number of liveborn children (fertility) and number of liveborn children that died before 5 years of age were recorded. Child mortality was then calculated as the proportion of liveborn children who had died by 5 years of age. As for the Agta, since total fertility was positively associated (*r* = 0.676, *n* = 45; *p* < 0.0001) with father's age at the time of data collection, residuals from this linear association were used in subsequent analyses as a measure of fertility corrected for age. Also, in light of a trend towards older individuals experiencing greater child mortality (*r* = 0.276, *n =* 36, *p* = 0.10) this was also corrected for year of birth during the study period using a similar method to control for possible age and cohort effects.

### Facial appearance ratings

Faces were all front on and photographed under natural lighting condition; faces were masked, cropped and resized as above. These images were then rated by 14 British observers (7 female; aged 18–24 years) and 9 Latin American observers (from Argentina, Belize, El Salvador and Guatemala; 6 female; aged 26–34) in an identical manner to the Agta stimuli with order of face presentation, and order of trait presentation randomized. As before, British observers were tested in person, while Latin American observers were recruited through email word of mouth and tested online. Again, one British observer was removed due to frequent negative correlations with other observers; otherwise inter-rater reliability was generally good (Attractiveness UK: α = .82; Masculinity UK: α = .84; Attractiveness Latin American: α = .64; Masculinity Latin American: α = .76) and British and Latin American scores were correlated (attractiveness: *r* = 0.709, *n* = 35; *p* < 0.001; masculinity: *r* = 0.619, *n* = 35; *p* < 0.001), so means were calculated based on all 22 raters. Both attractiveness and masculinity ratings were normally distributed (Shapiro Wilk’s > 0.9, *p* > 0.2 for both).

Mean age at the time of being photographed was 41.1 years (± 16.9) and the age of individuals in their photographs was negatively associated with ratings of attractiveness (*r* = -0.619, *n* = 35; *p* < 0.001) but positively associated with ratings of masculinity (*r* = 0.339, *n* = 35; *p* < 0.05)–i.e. older individuals looked less attractive and more masculine. Therefore scores were corrected for age at the time of photography by using regression residuals as measures in subsequent analyses.

### Facial appearance and offspring outcomes

**Fig 2:**
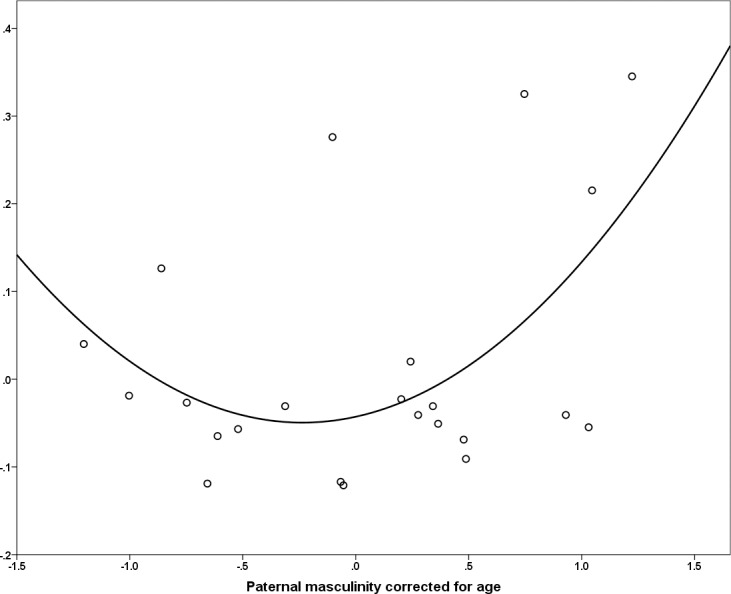
Facial masculinity and offspring mortality in Maya males. Quadratic association in Maya males [*F*(2,20) = 3.56, *p* < 0.05, *R*^2^ = .26] between facial masculinity corrected for age and offspring mortality (proportion dead before 5 years of age) corrected for age. Variables are residuals from masculinity-age and mortality-age regression analyses.

There was no relationship between masculinity and attractiveness (*r =* 0.044, *n* = 35, *p* = 0.800) corrected for age. Neither facial attractiveness (*r* = -0.069, *n* = 35, *p* = 0.67), nor masculinity (*r* = -0.061, *n* = 35, *p* = 0.73) was significantly associated with fertility and neither attractiveness (*r* = 0.080, *n* = 23, *p* = 0.72) or masculinity (*r* = 0.314, *n* = 23, *p* = 0.14) was significantly associated with offspring mortality (see Table 1 for data showing that aside from age, there were likewise no differences between fathers and non-fathers). However, again there was a significant quadratic relationship between offspring mortality and rated masculinity (Fig 2) such that the greatest offspring mortality occurred at the higher and lower ends of the masculinity spectrum, [*F*(2,20) = 3.56, *p* < 0.05, *R*^2^ = .26]. Although this initial model had residuals which were not normally distributed, excluding two outliers rendered the residuals normal without changing the nature of the quadratic result found; the original model was therefore retained. There were no other significant quadratic associations between facial appearance and offspring outcomes (either fertility or mortality).

Regarding potential mediating variables, although there was a association between total income and age-corrected fertility amongst the full Maya sample (*r* = .360, *n* = 56, *p* < .05) income was not correlated with age-corrected masculinity (*r* = -.168, *n* = 35, *p* = .335) or child mortality (*r* = .174, *n* = 36, *p* = .310) and there was no evidence of any non-linear relationships which could explain our results. Neither number of wives nor number of sexual partners was associated with age-corrected masculinity (*r* = .023, *n* = 35, *p* = .896; *r* = -.069, *n* = 35, *p* = .695 respectively).

Additional analyses were performed examining the Central American female raters’ attractiveness ratings of the images to determine whether different results might be found when considering cross-sex attraction, on a more within-culture basis. As per [24] and [25], both mean and individual women’s ratings were considered. Aside from one women demonstrating a positive association between age-corrected attractiveness ratings and age-corrected masculinity ratings (i.e. one woman was more attracted to male faces she found more masculine; *r* = .36, *p* = .03), there were no significant correlations between the overall, or individual, attractiveness ratings and any of the outcome or mediating variables (all │*r*│ < .28; all *p* > .1; 56 correlations in total).

## Discussion

The primary aim of the current study was to assess whether facial masculinity in males was associated with offspring mortality in two traditional societies, in order to test key assumptions of immunocompetence-based explanations for female preferences for masculinity in male faces. In both the Agta of the Philippines, and the Maya of Belize, no significant linear relationship was found, but rather in both populations there was a quadratic association between paternal masculinity and offspring mortality such that both high and low levels of masculinity were associated with an increase in child mortality relative to levels of masculinity closer to the population mean.

Like Apicella and colleagues’ data on voice pitch [17], we found no evidence of a significant general positive association between paternal masculinity and offspring survival. The finding, that it is intermediate, rather than high, levels of masculinity which are associated with the best offspring survival outcomes is inconsistent with the proposition that women may select more masculine mates in order to confer heritable immunity on their offspring. It is, however, consistent with recent evidence that when individual faces are manipulated on masculinity, women’s preferences seem to suggest an active preference for average levels of masculinity [24,25] and with more general research suggesting that there may be underlying genetic benefits associated with facial averageness [26]. Furthermore, our results are consistent with Scott and colleagues’ [27] recent data suggesting that preferences for masculine male faces are in fact weaker in traditionally living populations than in Western industrial nations. These authors suggest that preference variation shown by Western women may in fact reflect learning of associations between traits such as aggression and masculinity; our data confirm that amongst some traditional populations at least, there would likely be no offspring-health benefit to learn about anyway.

Plausibly, as set out in the trade-off model [5] high levels of paternal masculinity could be associated with significant genetic benefits for offspring but may not lead to improved child health because paternal masculinity may be associated with reduced parental investment and care. In traditional societies, paternal investment may be an important determinant of child nutrition [28]. Moreover, more masculine males might be more inclined to neglect and/or desert offspring since in contemporary industrialized societies there is evidence of a positive association between male testosterone levels and marital breakdown [29,30]. Our data do not permit a full examination of both these possibilities, but regarding the latter it should be noted that our Agta analyses excluded child deaths known to be due to accidents or violence, so it seems relatively unlikely that paternal behavioural factors such as aggression may be driving the increase in mortality in higher masculinity males.

Also of note was the finding that more masculine men did not sire more offspring than less masculine men, in either population. This is particularly noteworthy since alternatives to immunocompetence-based explanations for the importance of male facial masculinity tend to rest upon the proposition that more masculine males may achieve higher reproductive success via male-male competition [31] and may also confer reproductive advantages to their sons [6,8]. In addition to the lack of an association between masculinity and offspring numbers, more attractive individuals also did not sire more (or healthier) offspring. These combined results contrast with the data on vocal masculinity from the Hadza [17] and also with some data from modern populations (facial dominance [32]; facial attractiveness [33]). They are however, consistent with Silva and colleagues’ [34] data showing no relationship between facial attractiveness and fertility in either Senegalese or American men.

The lack of an association between male facial appearance and offspring number may be unsurprising in populations with more stable pair bonds as our two samples display, and our data are also subject to reporting bias insofar as individuals may not report (or know of) extra-pair conceptions. Although it should be noted that the Agta data provide at least a putative mother and father for each individual in the population (and ergo ought to include extra-pair offspring), these reported parents are not subject to genetic confirmation. Nevertheless, we have been unable to find evidence for direct positive sexual selection on male facial masculinity via mating or reproductive success. To shed further light on the issue, it is important that further research is conducted in traditional societies with higher known rates of extra-pair paternity and/or polygyny, in order to confirm whether or not our results are constrained by stable monogamy in our samples. It would also be useful to gather data on attractiveness ratings from individuals living under more similar circumstances to our populations; doing so in person would also circumvent the difficulties experienced here in recruiting a large enough sample of Filipino raters over the internet, in order to allow both within and across ethnicity attractiveness to be considered separately.

Finally it is important to acknowledge that our data are based on images of varying quality; while the Maya photos were taken in a systematic manner (albeit subject to the constraints of fieldwork and not in a laboratory), the Agta images were considerably more variable and less controlled in terms of pose, expression, lighting and even colour. Having found similar results in both samples, despite these weaknesses of the Agta images, increases the likelihood that a genuine underlying relationship (or lack of) has been detected here. However, we strongly recommend replication with more controlled images; use of a sample where controlled images existed of both male and female faces would furthermore allow researchers to calculate morphometric dimorphism (masculinity) of faces and avoid the need to rely on subjective ratings as in the current study (although we note that stimuli constructed based on rated masculinity yield comparable patterns of preferences amongst women as stimuli constructed based on objective sexual dimorphism; [35]).

Overall, we have examined the relationship between paternal facial features and child survival outcomes for the first time, and found no evidence to support the hypothesis that greater male masculinity (or attractiveness) is associated with greater offspring viability, and have rather found evidence to suggest that male masculinity may be subject to stabilising selection in this respect. In light of the small samples sizes used in the present study, and the possibility that, in principle, even small associations between appearance and offspring outcomes could have had important effects over long periods of time in ancestral environments, some caution is needed in interpreting the findings. However, given the possible implications of these results for a large literature examining female mate preferences for male masculinity, it is essential that our study is replicated, with larger samples, in other populations–particularly those practicing polygyny or with high rates of serial monogamy–and utilising genetic methods to confirm paternity.
